# Crystal structure and Hirshfeld surface analysis of 1,2,4-triazolium hydrogen oxalate

**DOI:** 10.1107/S2056989019017304

**Published:** 2020-01-07

**Authors:** Nutcha Ponjan, Purita Aroonchat, Kittipong Chainok

**Affiliations:** a Materials and Textile Technology, Faculty of Science and Technology, Thammasat University, Khlong Luang, Pathum Thani, 12121, Thailand; b Science Classroom in University-Affiliated School Projects (SCiUS), Suankularb, Wittayalai Rangsit School, Muang, Pathum Thani 12120, Thailand

**Keywords:** crystal structure, salts, hydrogen bonds, Hirshfeld surface

## Abstract

The asymmetric unit of the title 1:1 salt 1,2,4-triazolium hydrogen oxalate, C_2_H_4_N_3_^+^·C_2_HO_4_^−^ (**I**), comprises one 1,2,4-triazolium cation and one hydrogen oxalate anion. In the crystal, the hydrogen oxalate anions are linked by O—H⋯O hydrogen bonds into chains running parallel to [100]. In turn, the anionic chains are linked through the 1,2,4-triazolium cations by charge-assisted ^+^N—H⋯O^−^ hydrogen bonds into sheets aligned parallel to (01). The sheets are further stacked through π–π inter­actions between the 1,2,4-triazolium rings [centroid-to-centroid distance = 3.642 (3) Å, normal distance = 3.225 (3) Å, slippage 1.691 Å], resulting in the formation of a three-dimensional supra­molecular network. Hirshfeld surface analysis of the title salt suggests that the most significant contributions to the crystal packing are by H⋯O/O⋯H and H⋯N/N⋯H contacts involving the hydrogen bonds.

## Abstract 2

Charge-assisted N—H⋯O and O—H⋯O hydrogen bonds along with π–π inter­actions stabilize the crystalline state. Inter­molecular inter­actions are qu­anti­fied by Hirshfeld surface analysis.

## Chemical context   

The oxalate anion (C_2_O_4_^2–^), *i.e.* the complete deprotonation product of oxalic acid (C_2_H_2_O_4_), is a small, rigid, planar species and has been widely used as a ligand in the formation of coordination polymers (Gruselle *et al.*, 2006 ▸; Abraham *et al.*, 2014 ▸). This ligand possesses four electron-donating O atoms and can display versatile coordination modes upon metal complexation. As a result, a large number of compounds with multi-dimensional coordination networks with short inter­metallic distances have been synthesized along with the investigation of inter­esting properties (Clemente-León *et al.*, 2011 ▸). During our synthetic efforts to develop novel lanthanide coordination polymers with rigid, short, organic ligands including the oxalate anion, the title salt C_2_H_4_N_3_^+^·C_2_HO_4_^−^ (**I**) was obtained unexpectedly from the reaction of terbium(III) chloride hexa­hydrate, oxalic acid, and 1,2,4-triazole in water at room temperature.
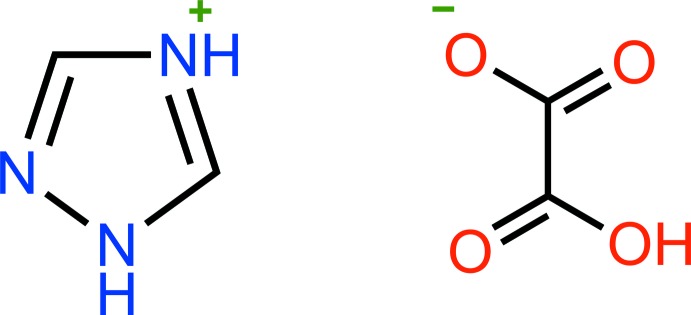


Herein, we describe the crystal structure and Hirshfeld surface analysis of the title salt (**I**).

## Structural commentary   

**Figure 1:**
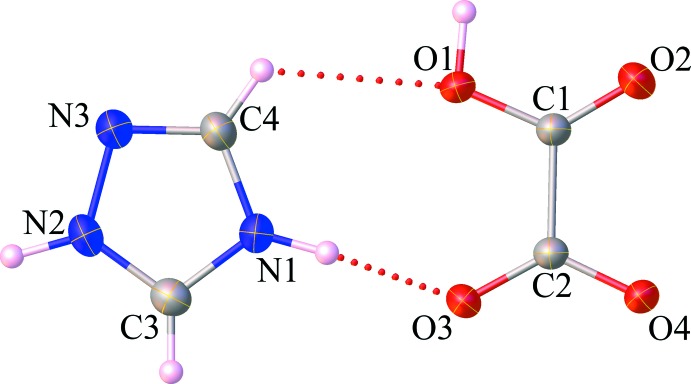
The structures of the mol­ecular entities in the title salt (**I**) showing the atom-labelling scheme. Displacement ellipsoids are drawn at the 50% probability level, and hydrogen bonds are shown as dotted lines.

As shown in Fig. 1 ▸, the asymmetric unit consists of one 1,2,4-triazolium cation and one hydrogen oxalate anion. In the hydrogen oxalate anion, the C1—O1 bond to the O atom that carries the H atom is significantly longer [1.3066 (14) Å] than the C1—O2 bond [1.1976 (15) Å], whereas the C2—O3 and C2—O4 bond lengths of the carboxyl­ate group show inter­mediate values [1.2370 (15) and 1.2586 (14) Å, respectively]. The hydrogen oxalate mol­ecule is nearly planar with an O2—C1—C2—O4 torsion angle of 2.3 (2)°. The 1,2,4-triazolium mol­ecule is perfectly planar with a root-mean-square (r.m.s.) deviation (excluding hydrogen atoms) of 0.001 Å. The cationic and anionic mol­ecules are coplanar with an r.m.s. deviation of 0.020 Å.

## Supra­molecular features   

**Figure 2:**
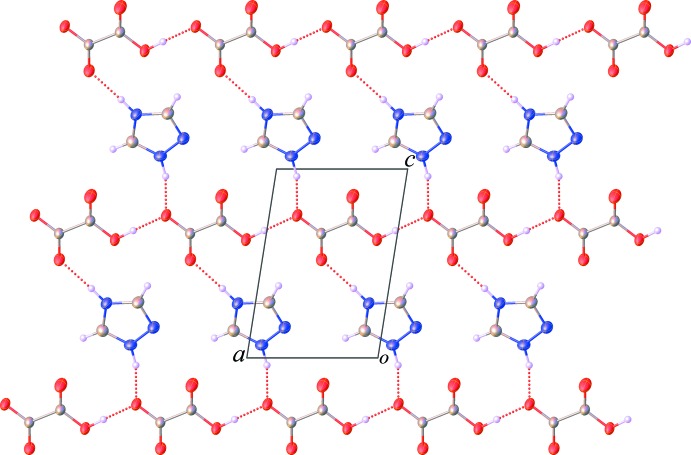
Partial view along [010] of the title salt (**I**), showing the O—H⋯O and N—H⋯O hydrogen-bonded sheet propagating parallel to (01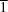). C—H⋯O and C—H⋯N hydrogen bonds are omitted for clarity.

**Figure 3:**
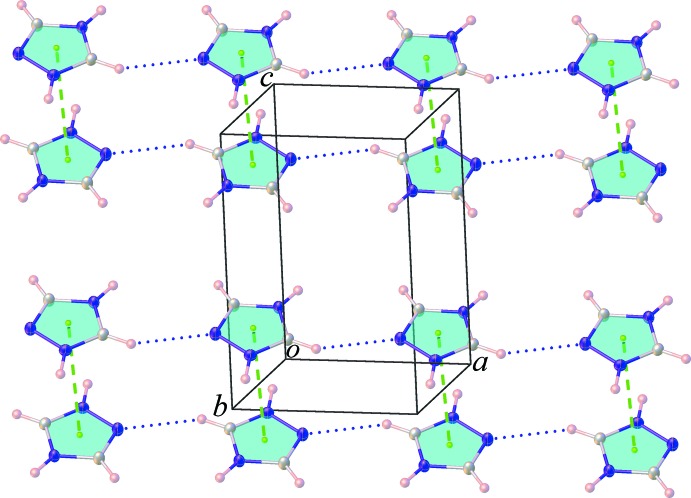
A view of the π–π stacking inter­actions along with the C—H⋯N hydrogen bonds in the title salt (**I**).

Extensive hydrogen-bonding inter­actions in the crystal of the title salt (**I**) are observed, the numerical values of which are collated in Table 1 ▸. As shown in Fig. 2 ▸, each hydrogen oxalate anion is linked with another anion by O—H⋯O hydrogen bonds into an infinite chain running parallel to [100]. The anionic chains are linked by charge-assisted ^+^N—H⋯O^−^ hydrogen bonds involving the 1,2,4-triazolium cations into sheets extending parallel to (01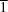). Additionally, intra­sheet C—H⋯O hydrogen and C—H⋯N hydrogen bonds involving the cationic mol­ecules are also observed. The sheets are further stacked through π–π inter­actions between the 1,2,4-triazolium rings [centroid-to-centroid distance = 3.642 (3) Å, normal distance = 3.225 (3) Å, slippage 1.691 Å], Fig. 3 ▸, resulting in the formation of a three-dimensional supra­molecular network.

## Hirshfeld surface analysis   

**Figure 4:**
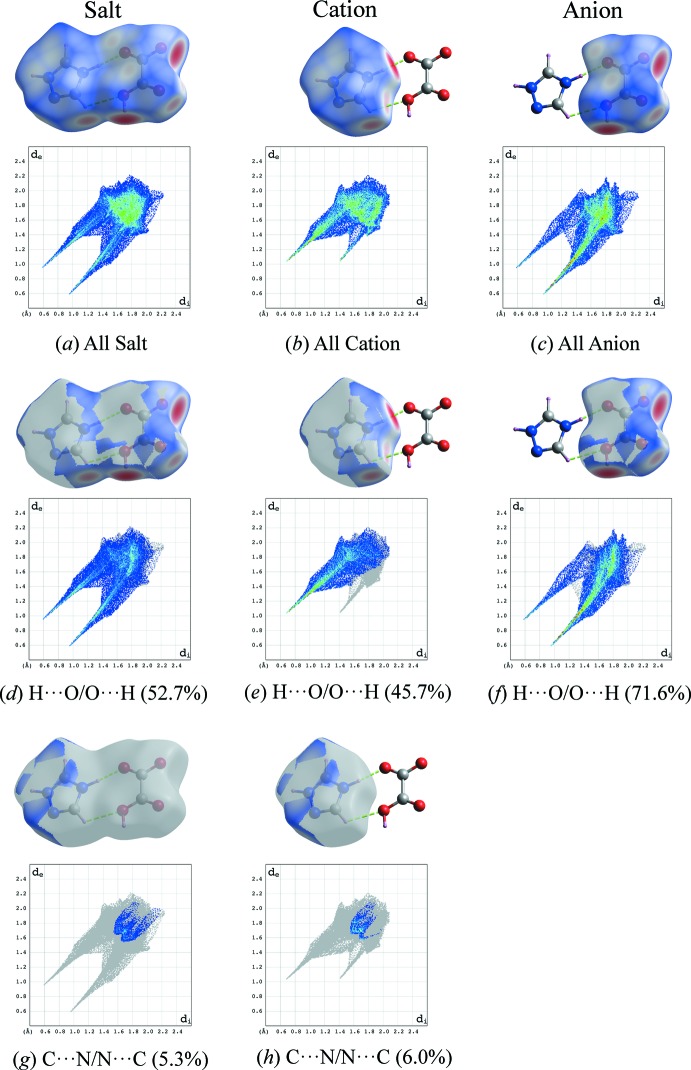
Full two-dimensional fingerprint plots of the title salt (**I**) (*a*), and its cation (*b*) and anion (*c*); separate contact types for the salt are given in (*d*)–(*h*) with relative contributions. Hirshfeld surfaces mapped over *d*_norm_ are displayed in all plots.

In order to qu­antify the nature of the inter­molecular inter­actions present in the crystal structure, Hirshfeld surfaces (McKinnon *et al.*, 2007 ▸) and their associated two-dimensional fingerprint plots (Spackman & McKinnon, 2002 ▸) were calculated using *CrystalExplorer17* (Turner *et al.*, 2017 ▸). The contribution of inter­atomic contacts to the *d*_norm_ surface of the title salt and the individual cations and anions are compared and shown in Fig. 4 ▸. In all cases, H⋯O/O⋯H contacts (*i.e.* ^+^N—H⋯O^−^, O—H⋯O, C–H⋯O) were found to be the major contributors towards the Hirshfeld surface, whereas H⋯N/N⋯H contacts (*i.e.* C—H⋯N) between the 1,2,4-triazolium cations play a minor role in the stabilization of the crystal packing. The differences between the individual fingerprints of cations and anions result from different distributions of the C⋯N/N⋯C contacts (*i.e.* π–π stacking). It was found that the H⋯H contacts have a relatively small contribution of only 7.7% to the entire Hirshfeld surfaces of the title salt.

## Database survey   

A search of the Cambridge Structural Database (CSD version 5.40, August 2019 update; Groom *et al.*, 2016 ▸) for structures with hydrogen oxalate gave 666 hits of which five are hydrogen-bonded salts of triazolium, *viz*. AFIVAO (Essid *et al.*, 2013 ▸) and CIRXEH (Matulková *et al.*, 2008 ▸), or imidazolium, *viz*. EVAPEX (Zhu, 2011 ▸), MEQPAZ (MacDonald *et al.*, 2001 ▸) and MEQPAZ01 (Prasad *et al.*, 2002 ▸).

## Synthesis and crystallization   

An aqueous solution (5 ml) of oxalic acid (0.09 g, 0.01 mol) and 1,2,4-triazole (0.07 g, 0.01 mmol) was added dropwise to an aqueous solution (5 ml) of TbCl_3_·6H_2_O (0.37 g, 0.01 mol) under constant stirring for one h. The resulting solution was filtered to remove any undissolved solid. The filtrate was allowed to slowly evaporate at room temperature. After two weeks, colourless block-shaped crystals of the title salt (**I**) suitable for X-ray analysis were obtained.

## Refinement   

Crystal data, data collection and structure refinement details are summarized in Table 2 ▸. The carboxyl and triazolium H atoms were located in difference-Fourier maps and were freely refined. Carbon-bound H atoms were placed in calculated positions and refined using a riding-model approximation with C—H = 0.93 Å and *U*_iso_(H) = 1.2*U*_eq_(C).

## Supplementary Material

Crystal structure: contains datablock(s) I. DOI: 10.1107/S2056989019017304/wm5529sup1.cif

Structure factors: contains datablock(s) I. DOI: 10.1107/S2056989019017304/wm5529Isup2.hkl

Supporting information file. DOI: 10.1107/S2056989019017304/wm5529Isup3.cdx

Supporting information file. DOI: 10.1107/S2056989019017304/wm5529Isup4.cml

CCDC reference: 1974526

Additional supporting information:  crystallographic information; 3D view; checkCIF report

## Figures and Tables

**Table 1:** Hydrogen-bond geometry (Å, °)

*D*—H⋯*A*	*D*—H	H⋯*A*	*D*⋯*A*	*D*—H⋯*A*
O1—H1*A*⋯O4^i^	0.94 (2)	1.61 (2)	2.5447 (13)	175.0 (18)
N1—H1⋯O3	0.91 (2)	1.81 (2)	2.7199 (15)	175.4 (18)
N2—H2⋯O4^ii^	0.96 (2)	1.80 (2)	2.7443 (15)	167.3 (19)
C3—H3⋯O2^iii^	0.93	2.40	3.1717 (17)	141
C3—H3⋯N3^iv^	0.93	2.58	3.3939 (18)	146
C4—H4⋯O1	0.93	2.45	3.0289 (16)	120
C4—H4⋯O3^i^	0.93	2.30	3.1625 (17)	153

Symmetry codes: (i) 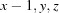; (ii) 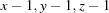; (iii) 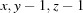; (iv) 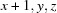.

**Table 2:** Experimental details

Crystal data	
Chemical formula	C_2_H_4_N_3_^+^·C_2_HO_4_^−^
*M*_r_	159.11
Crystal system, space group	Triclinic, *P* 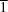
Temperature (K)	296
*a*, *b*, *c* (Å)	5.592 (1), 7.2162 (12), 8.4021 (13)
α, β, γ (°)	109.148 (6), 93.889 (7), 103.282 (6)
*V* (Å^3^)	307.92 (9)
*Z*	2
Radiation type	Mo *K*α
μ (mm^−1^)	0.16
Crystal size (mm)	0.34 × 0.22 × 0.22

Data collection	
Diffractometer	Bruker D8 Quest CMOS PHOTON II
Absorption correction	Multi-scan (*SADABS*; Bruker, 2016 ▸)
*T*_min_, *T*_max_	0.638, 0.746
No. of measured, independent and observed [*I* > 2σ(*I*)] reflections	4805, 1524, 1278
*R*_int_	0.038
(sin θ/λ)_max_ (Å^−1^)	0.668

Refinement	
*R*[*F* ^2^ > 2σ(*F* ^2^)], *wR*(*F* ^2^), *S*	0.037, 0.102, 1.07
No. of reflections	1524
No. of parameters	112
H-atom treatment	H atoms treated by a mixture of independent and constrained refinement
Δρ_max_, Δρ_min_ (e Å^−3^)	0.37, −0.20

Computer programs: *APEX3* and *SAINT* (Bruker, 2016 ▸), *SHELXT* (Sheldrick, 2015*a* ▸), *SHELXL* (Sheldrick, 2015*b* ▸) and *OLEX2* (Dolomanov *et al.*, 2009 ▸).

## supplementary crystallographic information

### Crystal data

**Table:**

C_2_H_4_N_3_^+^·C_2_HO_4_^−^	*Z* = 2
*M**_r_* = 159.11	*F*(000) = 164
Triclinic, *P*1	*D*_x_ = 1.716 Mg m^−^^3^
*a* = 5.592 (1) Å	Mo *K*α radiation, λ = 0.71073 Å
*b* = 7.2162 (12) Å	Cell parameters from 2650 reflections
*c* = 8.4021 (13) Å	θ = 2.6–28.3°
α = 109.148 (6)°	µ = 0.16 mm^−^^1^
β = 93.889 (7)°	*T* = 296 K
γ = 103.282 (6)°	Block, light colourless
*V* = 307.92 (9) Å^3^	0.34 × 0.22 × 0.22 mm

### Data collection

**Table:**

Bruker D8 Quest CMOS PHOTON II diffractometer	1524 independent reflections
Radiation source: sealed x-ray tube	1278 reflections with *I* > 2σ(*I*)
Graphite monochromator	*R*_int_ = 0.038
Detector resolution: 7.39 pixels mm^-1^	θ_max_ = 28.4°, θ_min_ = 2.6°
ω and φ scans	*h* = −6→7
Absorption correction: multi-scan (*SADABS*; Bruker, 2016)	*k* = −9→8
*T*_min_ = 0.638, *T*_max_ = 0.746	*l* = −11→11
4805 measured reflections	

### Refinement

**Table:**

Refinement on *F*^2^	0 restraints
Least-squares matrix: full	Hydrogen site location: mixed
*R*[*F*^2^ > 2σ(*F*^2^)] = 0.037	H atoms treated by a mixture of independent and constrained refinement
*wR*(*F*^2^) = 0.102	*w* = 1/[σ^2^(*F*_o_^2^) + (0.0441*P*)^2^ + 0.0686*P*] where *P* = (*F*_o_^2^ + 2*F*_c_^2^)/3
*S* = 1.07	(Δ/σ)_max_ < 0.001
1524 reflections	Δρ_max_ = 0.37 e Å^−^^3^
112 parameters	Δρ_min_ = −0.20 e Å^−^^3^

### Special details

**Table:**

Geometry. All esds (except the esd in the dihedral angle between two l.s. planes) are estimated using the full covariance matrix. The cell esds are taken into account individually in the estimation of esds in distances, angles and torsion angles; correlations between esds in cell parameters are only used when they are defined by crystal symmetry. An approximate (isotropic) treatment of cell esds is used for estimating esds involving l.s. planes.

### Fractional atomic coordinates and isotropic or equivalent isotropic displacement parameters (Å^2^)

**Table:**

	*x*	*y*	*z*	*U*_iso_*/*U*_eq_	
O1	0.14792 (16)	0.88282 (15)	0.62587 (12)	0.0335 (3)	
H1A	0.020 (4)	0.935 (3)	0.674 (2)	0.069 (6)*	
O2	0.38612 (19)	1.12875 (18)	0.85543 (14)	0.0506 (3)	
O3	0.54794 (17)	0.76994 (15)	0.51985 (12)	0.0365 (3)	
O4	0.78723 (16)	1.00344 (15)	0.75427 (12)	0.0345 (3)	
N1	0.1358 (2)	0.53625 (17)	0.28813 (14)	0.0305 (3)	
H1	0.270 (4)	0.620 (3)	0.367 (2)	0.057 (5)*	
N2	−0.0976 (2)	0.30879 (17)	0.06817 (14)	0.0305 (3)	
H2	−0.162 (4)	0.201 (3)	−0.039 (3)	0.063 (6)*	
N3	−0.2527 (2)	0.40637 (18)	0.16122 (14)	0.0352 (3)	
C1	0.3594 (2)	0.98777 (18)	0.72504 (15)	0.0264 (3)	
C2	0.5818 (2)	0.91101 (18)	0.65830 (15)	0.0249 (3)	
C3	0.1335 (2)	0.38679 (19)	0.14421 (16)	0.0304 (3)	
H3	0.270783	0.345169	0.104695	0.036*	
C4	−0.1040 (2)	0.5435 (2)	0.29349 (17)	0.0340 (3)	
H4	−0.155357	0.635686	0.381391	0.041*	

### Atomic displacement parameters (Å^2^)

**Table:**

	*U*^11^	*U*^22^	*U*^33^	*U*^12^	*U*^13^	*U*^23^
O1	0.0174 (4)	0.0398 (5)	0.0303 (5)	0.0095 (4)	0.0014 (4)	−0.0054 (4)
O2	0.0280 (5)	0.0571 (7)	0.0392 (6)	0.0155 (5)	−0.0002 (4)	−0.0201 (5)
O3	0.0224 (5)	0.0396 (5)	0.0306 (5)	0.0093 (4)	0.0027 (4)	−0.0101 (4)
O4	0.0183 (4)	0.0414 (5)	0.0294 (5)	0.0093 (4)	−0.0003 (4)	−0.0064 (4)
N1	0.0231 (5)	0.0316 (6)	0.0269 (5)	0.0052 (4)	−0.0002 (4)	−0.0003 (4)
N2	0.0319 (6)	0.0286 (5)	0.0225 (5)	0.0072 (4)	0.0021 (4)	−0.0009 (4)
N3	0.0264 (6)	0.0380 (6)	0.0294 (6)	0.0091 (5)	0.0012 (5)	−0.0029 (5)
C1	0.0190 (6)	0.0304 (6)	0.0236 (6)	0.0083 (5)	0.0023 (4)	0.0004 (5)
C2	0.0183 (5)	0.0274 (6)	0.0238 (6)	0.0071 (4)	0.0033 (4)	0.0018 (5)
C3	0.0281 (6)	0.0313 (6)	0.0285 (6)	0.0103 (5)	0.0059 (5)	0.0046 (5)
C4	0.0273 (7)	0.0359 (7)	0.0275 (6)	0.0101 (5)	0.0014 (5)	−0.0038 (5)

### Geometric parameters (Å, º)

**Table:**

O1—H1A	0.94 (2)	N2—H2	0.96 (2)
O1—C1	1.3066 (14)	N2—N3	1.3677 (16)
O2—C1	1.1976 (15)	N2—C3	1.3089 (17)
O3—C2	1.2370 (15)	N3—C4	1.2967 (17)
O4—C2	1.2586 (14)	C1—C2	1.5413 (17)
N1—H1	0.91 (2)	C3—H3	0.9300
N1—C3	1.3272 (16)	C4—H4	0.9300
N1—C4	1.3568 (18)		
			
C1—O1—H1A	109.1 (12)	O2—C1—C2	121.49 (11)
C3—N1—H1	127.8 (13)	O3—C2—O4	125.86 (11)
C3—N1—C4	106.09 (11)	O3—C2—C1	119.53 (11)
C4—N1—H1	126.1 (13)	O4—C2—C1	114.60 (10)
N3—N2—H2	120.4 (13)	N1—C3—H3	126.3
C3—N2—H2	128.5 (13)	N2—C3—N1	107.36 (12)
C3—N2—N3	111.11 (11)	N2—C3—H3	126.3
C4—N3—N2	103.62 (11)	N1—C4—H4	124.1
O1—C1—C2	112.90 (10)	N3—C4—N1	111.81 (12)
O2—C1—O1	125.61 (12)	N3—C4—H4	124.1
			
O1—C1—C2—O3	3.15 (18)	N3—N2—C3—N1	0.08 (15)
O1—C1—C2—O4	−177.26 (11)	C3—N1—C4—N3	0.23 (17)
O2—C1—C2—O3	−177.27 (13)	C3—N2—N3—C4	0.06 (15)
O2—C1—C2—O4	2.3 (2)	C4—N1—C3—N2	−0.18 (15)
N2—N3—C4—N1	−0.18 (16)		

### Hydrogen-bond geometry (Å, º)

**Table:**

*D*—H···*A*	*D*—H	H···*A*	*D*···*A*	*D*—H···*A*
O1—H1*A*···O4^i^	0.94 (2)	1.61 (2)	2.5447 (13)	175.0 (18)
N1—H1···O3	0.91 (2)	1.81 (2)	2.7199 (15)	175.4 (18)
N2—H2···O4^ii^	0.96 (2)	1.80 (2)	2.7443 (15)	167.3 (19)
C3—H3···O2^iii^	0.93	2.40	3.1717 (17)	141
C3—H3···N3^iv^	0.93	2.58	3.3939 (18)	146
C4—H4···O1	0.93	2.45	3.0289 (16)	120
C4—H4···O3^i^	0.93	2.30	3.1625 (17)	153

Symmetry codes: (i) *x*−1, *y*, *z*; (ii) *x*−1, *y*−1, *z*−1; (iii) *x*, *y*−1, *z*−1; (iv) *x*+1, *y*, *z*.
